# Incomplete sclerotization and phylogeny: The phylogenetic classification of *Plastocerus* (Coleoptera: Elateroidea)

**DOI:** 10.1371/journal.pone.0194026

**Published:** 2018-03-14

**Authors:** Ladislav Bocak, Michal Motyka, Matej Bocek, Milada Bocakova

**Affiliations:** Department of Zoology, Faculty of Science, Palacky University, Olomouc, Czech Republic; National Cheng Kung University, TAIWAN

## Abstract

The relationships of the monogeneric family Plastoceridae Crowson, 1972 (Coleoptera: Elateroidea) have remained contentious due to its modified morphology, incorrect information on incomplete metamorphosis of females and the absence of molecular data. We produced the sequences for *P*. *angulosus* (Germar, 1844) (the type-species of *Plastocerus* Schaum, 1852) and performed molecular phylogenetic analyses to estimate its position. The analyses of Elateroidea (186 spp.) and Elateridae (110 spp.) molecular datasets of two mitochondrial and two nuclear gene fragments repeatedly placed *Plastocerus* Schaum, 1852 in relationships with the elaterid genera *Oxynopterus* Hope, 1842 and *Pectocera* Hope, 1842. Alternative topologies were rejected by likelihood tests. Therefore, Plastoceridae Crowson, 1972 are down-ranked to the subfamily Plastocerinae in Elateridae Leach, 1815. We suggest that the morphology-based placement and high rank for some elateroid lineages were inferred from the presence of homoplasies which evolved due to incomplete sclerotization. Distantly related soft-bodied elateroids share freely movable and transverse coxae, a shortened prosternum, and a weakly sclerotized abdomen with free ventrites. Importantly, the apomorphic structures characteristic for their closest relatives, such as the prosternal process, mesoventral cavity, and intercoxal keel in the first abdominal ventrite are regularly absent. Consequently, morphology-based phylogenetic analyses suggest deeply rooted positions for lineages without expressed apomorphic character states. Molecular data represent an independent character system that is not affected by the convergent morphological evolution, and therefore molecular phylogenies can elucidate the relationships of incompletely sclerotized lineages.

## Introduction

A large body of recent work has been devoted to beetle molecular phylogenetics and the monophyly of most families, and their relationships are well-supported [1–3]. Nevertheless, some family-group taxa still maintain their original formal placement based solely on morphology due to the inaccessibility of specimens for DNA analyses [2,4]. Contrary to hyper-diverse and morphologically uniform lineages, such as phytophagous weevils, leaf beetles or jewel beetles, the Elateroidea is known for high morphological diversity. The superfamily contains well-sclerotized (i.e., Artematopodidae, Throscidae, Cerophytidae, Eucnemidae, and Elateridae) and soft-bodied lineages (i.e., Omethidae incl. Telegeusinae, Cantharidae, Lampyridae, Lycidae, Omalisidae, Phengodidae and Rhagophthalmidae). The soft-bodied lineages were first placed in the superfamily Cantharoidea [5] and later included in Elateroidea and hypothesized as a cantharoid clade [6,7]. Morphological and DNA-based analyses of Elateroidea provide conflicting phylogenetic hypotheses. The morphology-based analyses indicate the monophyly of Cantharoidea *sensu* Crowson [4–7]. Conversely, all molecular analyses have suggested distant relationships of various soft-bodied, superficially similar elateroid lineages [1–3,8–10].

The monophyly and relationships of major families are well-supported by various studies[1–3,8–10]. In contrast to these families, Elateroidea contained several species-poor families with poorly supported relationships, such as Drilidae Blanchard, 1845, Telegeusidae Leng, 1920, Cebrionidae Latreille, 1802, Podabrocephalidae Pic, 1930, Cneoglossidae Champion, 1897, and Plastoceridae Crowson, 1972 [11–13]. Except the last mentioned, all have already been down-ranked from the family level or excluded from Elateroidea [2,13–15]. Plastoceridae remain the last elateroid family with an unclear position.

The family Plastoceridae was proposed by Crowson [5] in Cantharoidea for *Plastocerus angulosus* (Germar, 1845) from Turkey and Greece (the type species of *Plastocerus* Schaum, 1852 nec Leconte, 1853) and *P*. *thoracicus* Fleutiaux, 1918 from Southeast Asia. Further genera, earlier placed in Plastoceridae as defined by Leconte [16] and Schwarz [17], were excluded due to their divergent morphology, and they are now placed in Elaterinae: Cebrionini [5,15]. Plastoceridae has been variously delimited, but it has kept the family rank for most of the time since their proposal [2,4,5,13,17–19]. The previous analyses were based exclusively on adult morphology and suggested a deeply rooted position that justified the family rank [5–7]. There is conflicting information about the female of *Plastocerus* in recent literature. Crowson [5] stated that the female resembles those of *Omalisus* Geoffroy, 1762, is wingless, and has shortened elytra. Branham [18] wrote that the female is unknown.

The aim of this study is to provide molecular evidence for the phylogenetic placement and formal classification of Plastoceridae. Exclusively using molecular data, we try to avoid the unwitting inclusion of convergent morphological characters in phylogenetic estimates which might be the result of parallel morphological evolution. Therefore, we separately discuss morphological traits that supported the relationships of Plastoceridae and cantharoid lineages. Additionally, we provide the first illustrations of a *Plastocerus* female.

## Material and methods

*Plastocerus angulosus* has recently been collected and fixed for DNA isolation. A single population was sampled in south-western Turkey (Canakkale province, Ayvacık district, Behramkale environ, 20 m a. s. l., 3 Jul. 2015, 39°29'N, 26°20'E). The voucher specimen is preserved in the voucher collection of the Department of Zoology, Palacky University, tr. 17 listopadu 50, 771 46 Olomouc, Czech Republic (curator L. Bocak, ladislav.bocak@upol.cz) and is designated by the voucher number UPOL A01544. We have not found any females in the field and only two dry-mounted female specimens from Turkey were identified in the collection of the Coleoptera Section in the Natural History Museum in London (curator Michael Geiser, locality data: "Besika Bay" (= Beşik Bay) and "Brussa" (= Bursa), no further data locality data available).

The male was used for DNA extraction; the laboratory procedures followed those reported by Bocakova *et al*. [14]. The newly produced sequences of *Plastocerus angulosus* were submitted to GenBank under numbers KX648440, 442, 444, and 446. All accession numbers are listed in Table A in S1 Supplements. The Elateroidea and *Plastocerus angulosus cox1* and *rrnL* mtDNA and nuclear *SSU* and *LSU* rRNA fragments were assembled in a single dataset representing 186 taxa (Tables A and B in S1 Supplements). All sequences were aligned using MAFFT 7.2 (Q-INS-I algorithm, default parameters; [20]); the protein coding fragments were checked for reading frames. The dataset contained numerous *SSU* and *LSU* fragment*s* with long insertions whose negative effect on the robustness of the alignment and phylogenetic analysis was described by Bocak *et al*. [9]. The rRNA sequences of Elateridae are known for the low proportion of length variable regions [9]. Therefore, we additionally compiled a four-gene dataset for the analysis of Elateridae (109 taxa) + *Plastocerus angulosus* to test, if the analysis of a different dataset recovers similar relationships (Table C in S1 Supplements). The sequences were aligned in the same way as described above. These two datasets substantially differ in the representation of taxa and length of the aligned sequences.

We estimated maximum likelihood (ML) trees using IQ-Tree 1.5.5 [21]. Both datasets were partitioned by genes, and substitution models were identified using ModelFinder [22]. Bootstrap branch support values were obtained applying ultrafast likelihood bootstrap [23] with 1000 replicates. Additionally, because *Plastocerus* was inferred to be in the cantharoid clade by morphological analyses [6,7], we evaluated the confidence of alternative phylogenetic relationship hypotheses using the approximately unbiased (AU) test [24], one sided Kishino-Hasegawa test (KH) [25] and Expected Likelihood Weight (ELW) [26]. The unconstrained ML tree was tested against topologies where *Plastocerus* was a sister to (A) Lycidae, (B) Cantharidae, (C) Omethidae, and (D) Lampyridae. All of them represent major cantharoid lineages that were earlier inferred to be related to Plastoceridae [4–7]. The constrained phylogenies were estimated in IQ-TREE using concatenated datasets and the same settings. All tests were performed in IQ-TREE [21] testing per site log likelihoods using the “-au” option and 10,000 bootstrap replicates.

## Results

The sequences of *SSU* rRNA, the D2 loop of *LSU* rRNA, *rrnL* mtDNA and *cox1* mtDNA were amplified from the adult male from Behramkale. These sequences were merged with earlier published data [8, 9,14]. The Elateroidea and Elateridae datasets included 186 taxa with 4923 homologous positions and 0.6% missing data and 110 taxa with 3560 homologous positions and 2.4% missing data, respectively (Table A in S1 Supplements). Information about the gene partitioning scheme, the best models and completeness of datasets is listed in Tables A and C in S1 Supplements.

**Fig 1:**
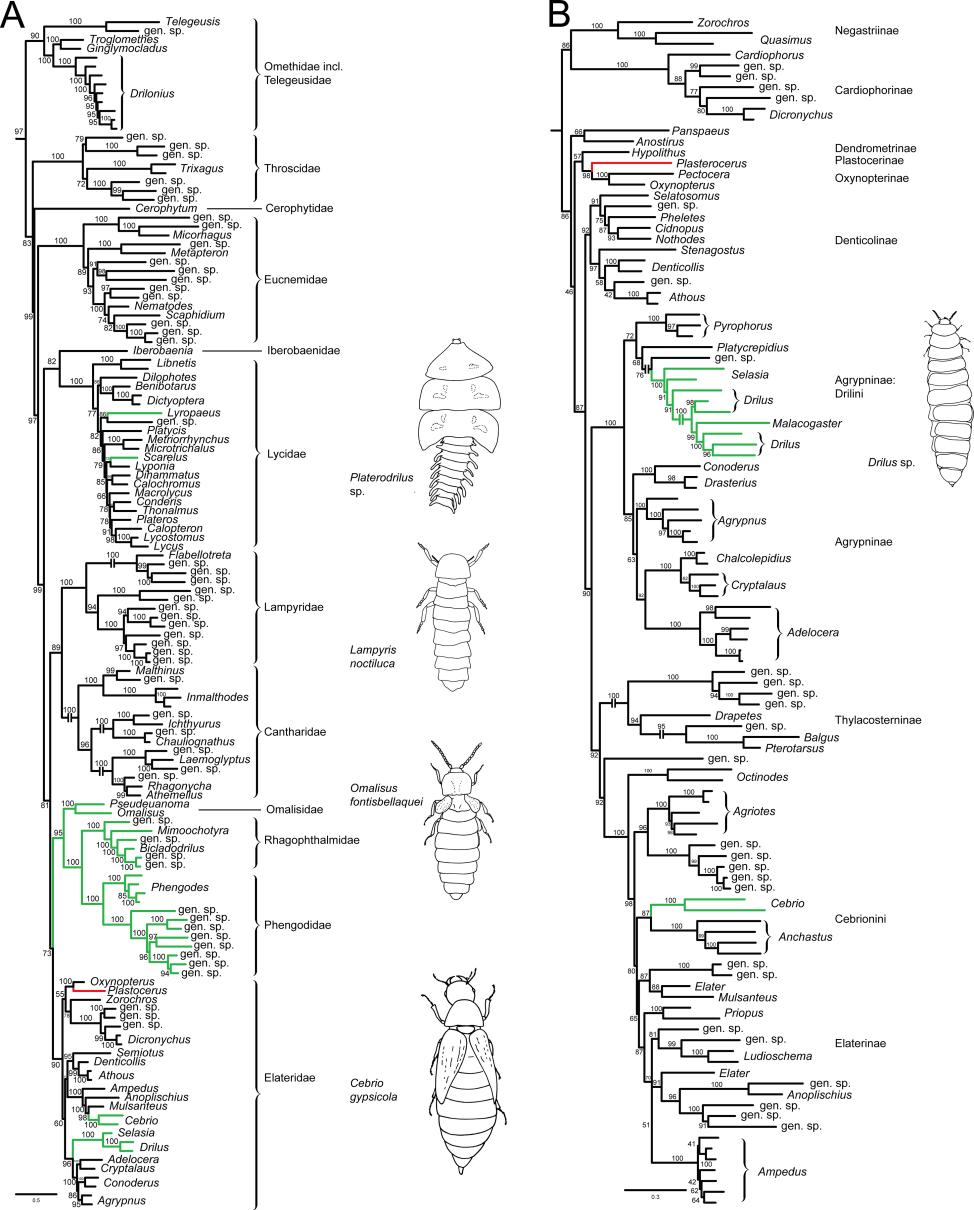
The phylogeny of Elateroidea and Elateridae. (A) The phylogenetic hypothesis inferred from the MAFFT-aligned Elateroidea dataset (all outgroups removed), branch labels designate bootstrap support values. (B) the phylogenetic hypothesis inferred from the MAFFT-aligned Elateridae dataset. Branch labels as above. The branches representing taxa with vestigial or absent female elytra are marked by green colour.

The analysis of the complete Elateroidea dataset recovered all families as monophyletic with high bootstrap (BS) and *Plastocerus angulosus* was recovered as a terminal branch within Elateridae in sister-relationships with *Oxynopterus* sp. (Fig 1A; BS 100%). The separate analyses of the Elateridae dataset confirmed previous placement, and *Plastocerus* was recovered in the same position, as a sister to the robustly supported *Oxynopterus* + *Pectocera* clade (BS 100%). The *Oxynopterus* + (*Plastocerus* + *Pectocera*) clade was inferred within the paraphyletic assemblage of Dendrometrinae taxa (Fig 1B). Moreover, likelihood scores of the ML constrained topologies with alternative *Plastocerus angulosus* placements were significantly worse than the ML score of the unconstrained tree (Table D in S1 Supplements).

## Discussion

Plastoceridae is one of a few beetle families that, has never been included in molecular analyses [2,3]. Here, the first molecular data were produced for molecular phylogenetic inference. In contrast with the earlier placement based on morphology [4–7,13,16–18], our analyses of the Elateroidea and Elateridae datasets consistently inferred *Plastocerus* as related to *Oxynopterus* sp. and *Pectocera* sp. (Fig 1A and 1B; Oxynopterinae or Oxynopterini [8]; BS 100% and 98%). Although the structure of Elateroidea and Elateridae alignments was different due the presence of sequences with long insertions in the first dataset, both analyses regularly recovered the *Oxynopterus* + *Plastocerus* clade. The BS values supporting the position of *Plastocerus* were higher than most of the BS values supporting relationships among tribe- or subfamily-rank elaterid clades in the current analyses. The *SSU* and *LSU* loop regions of Elateridae are short and almost length invariable in comparison with other elateroid families [9], and the analyses regularly provide a low support for the monophyly of Elateridae [8,14,15], or Elateridae were even not recovered as a monophylum in a deep conflict with morphology [2]. To test the robustness of the recovered placement within Elateridae, we compared the likelihood of the unconstrained topology to alternatives with *Plastocerus* placed in relationships with various cantharid families. All constrained topologies were significantly rejected.

We conclude that the position of *Plastocerus* in Elateridae is stable and sufficiently supported and we propose that Plastoceridae Crowson, 1972 are down-ranked to Plastocerinae in Elateridae Leach, 1815. Although our analyses indicate close relationships of *Plastocerus* and the clade represented by *Pectocera* and *Oxynopterus*, we prefer the subfamily rank as a conservative proposal until further data are available.

Based on morphology, *Plastocerus* has always been placed in the cantharoid lineage [4–7], but its relationships have only been investigated in a formal morphology-based phylogenetic analyses on two occasions. Lawrence [6] found *Plastocerus* as a sister-lineage to Cantharoidea, but individual cantharoid lineages were not coded, and all soft-bodied elateroids were replaced by a single composite taxon. Later, the family Plastoceridae was excluded from the subsequent re-analysis of the dataset [27]. The latest morphology-based analysis contained only families that were placed in Cantharoidea in that time [7], and *Plastocerus* was visualized as a sister-taxon to the remaining cantharoid taxa. Unfortunately, Lawrence *et al*. [4] did not include *Plastocerus* in the dataset. Their study represents the largest morphology-based analysis of beetle phylogeny, and the cantharoid clade was inferred to be in a terminal position within Elateroidea. The current classification of *Plastocerus* and the morphology-based topologies are in deep conflict with all molecular analyses (Fig 1) [1–3,8–10,14,15]. Therefore, we discuss the morphological characters supporting the close relationships of *Plastocerus* and cantharoid lineages.

### Adult morphology of *Plastocerus*

**Fig 2:**
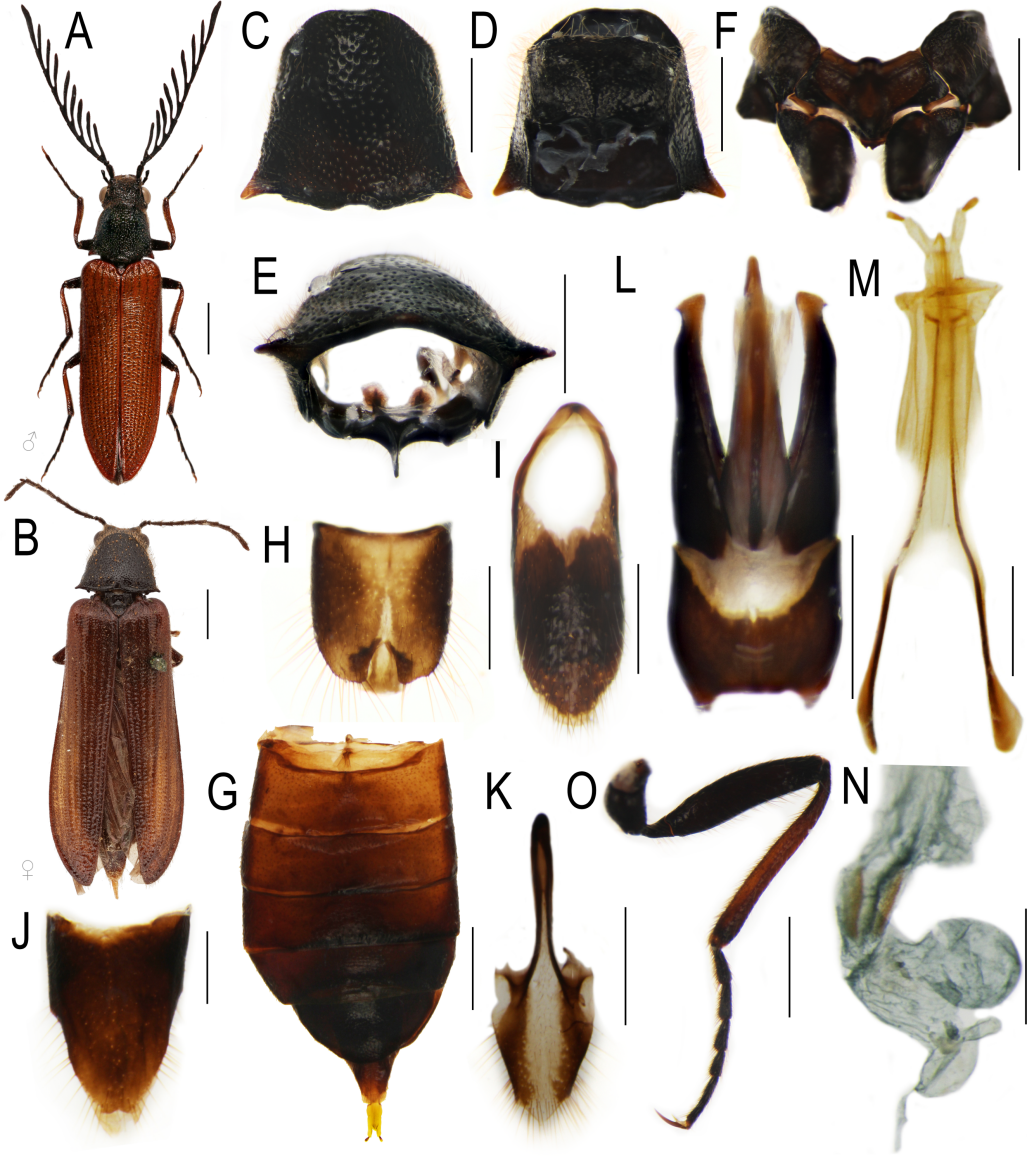
***Plastocerus angulosus* (Germar)** (A–B) general appearance, male and female; (C–E) prothorax, dorsal, ventral, and posterior view; (F) mesothorax, ventrally; (G) female abdomen, ventrally; (H–I) male terminal abdominal segments; (J–K) female terminal abdominal segments; (L) male genitalia; (M) ovipositor; (N) female sexual ducts; (O) hind leg, male. Scale 2 mm (A, B, G), 1 mm (C–F, J, K, M, O), 0.5 mm (H, I, L, N).

**Fig 3:**
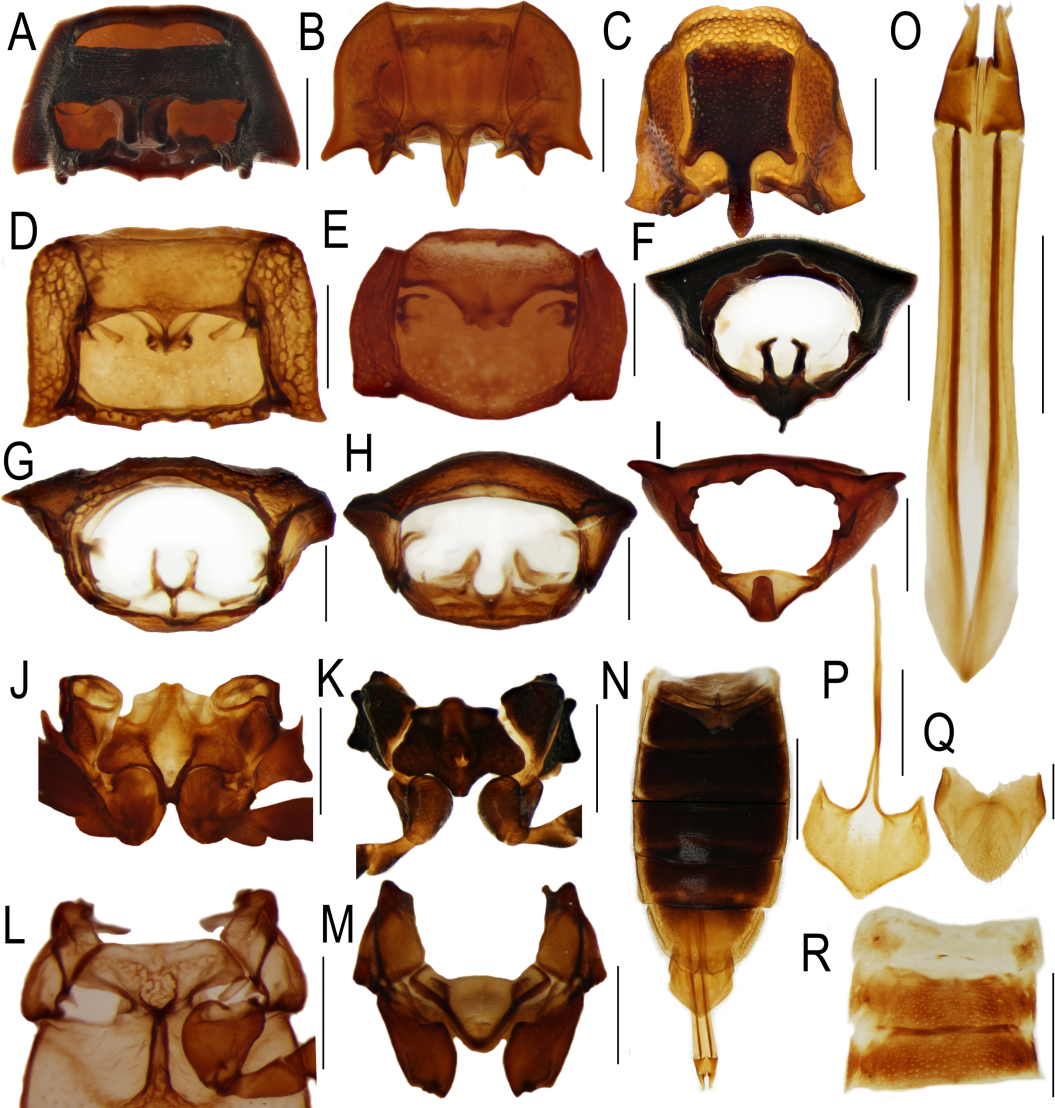
Morphology of Elateriformia. Prothorax, ventrally (A) Dascillidae, *Dascillus* sp., (B) Elateridae: Dimini, *Penia turnai* Schimmel, (C) Denticollini, *Denticollis linearis* (Linneaus), (D) Omalisidae: *Omalisus fontisbellaquei* (Geoffroy) (E) Elateridae: Drilini, *Drilus concolor* Ahrens. Pronotum, posterior view (F) Dascillus sp., (G) *Omalisus fontisbellaquei*, (H) *Drilus concolor*. (I) *Denticolis linearis*. Mesosternum (J) *Penia turnai*, (K) *Denticollis linearis*, (L) *Omalisus fontisbellaquei*, (M) *Drilus concolor*. *Denticollis linearis* (N) female, abdomen, ventrally, (O) ovipositor, (P–Q) female terminal abdominal segments. (R) *Drilus concolor*, basal abdominal ventrites. Scales 2 mm (N), 1 mm (A–C, F–K, O–R), 0.5 mm (D, E, L, M).

The body of *Plastocerus* is quite well-sclerotized (Fig 2A and 2B), and its general morphology differs only slightly when compared with the fully-sclerotized elateroids (Fig 3B, 3C, 3J, 3K, 3I and 3N). *Plastocerus angulosus* shares the following characters with Elateridae: the posterior angles of the pronotum are acutely projected (Fig 2C and 2D); the female abdomen has six visible sclerotized ventrites (Fig 2G, similar to Elateridae: *Denticollis*; Fig 3N, unlike Lycidae and Cantharidae, [28]); male genitalia are trilobate with outwardly hooked apexes of parameres (Fig 2L, similar to numerous Elateridae and Omalisidae: *Omalisus*, see [29]); and the female genitalia have very short coxites and long at least apically flattened valvifers (very similar to those of *Denticollis*, Fig 3O, but also Lycidae: Calochromini and Dictyopterini [29]).

*Plastocerus angulosus* differs from fully-sclerotized Elateridae in the absence of the promesothoracic interlocking mechanism (Fig 2D and 2E), in the transverse prosternum, transverse prothoracic coxae with exposed trochantins, freely articulated abdominal ventrites and a weakly developed intercoxal keel of the abdominal ventrite 1 (Fig 2K) [5]. These characters resemble structures known in the distantly related soft-bodied elateroids, i.e., the families placed in former Cantharoidea [5, 7]. The cantharoid taxa do not have a promesothoracic click mechanism, and their prosternal process is short (Omalisidae, Fig 3D) [30] or absent (Elateridae: Agrypninae: Drilini, Fig 3E). The prosternal process and the pair of internal prothoracic processes of *Plastocerus* are shortened and resembles those of Omalisidae (Fig 3F), Iberobaeniidae [10] and distantly related Dascillidae (Figs 2E, 3A and 3F). The prosternum of *Plastocerus* is only slightly wider than it is long (Fig 2C) and is never as long as in Elateridae (3B–C). The prosternum of soft-bodied elateroid lineages is regularly transverse and approximately two times wider than it is long in Omalisidae (Fig 3D) and Elateridae: Drilini (Fig 3E), or even shorter in Lycidae [28,31]. The mesosternites of various elateroid taxa are similar in their shape (Figs 2F and 3J–3M), but the taxa with a fully developed click mechanism have a clear depression where the prosternal process fits. This depression is inconspicuous or absent in the taxa without the click mechanism. The modifications which can be assigned to incomplete sclerotization can be further demonstrated in the abdominal morphology. Although the female abdomen of *Plastocerus* has all ventrites free, they are more sclerotized than in any typical soft-bodied elateroid taxon (Figs 2K and 3R [6,18]). The soft-bodied lineages also have all abdominal ventrites free, but their abdomen has very extensive membranes and often unclear limits of sclerites. Further, *Plastocerus* has only a short, weakly sclerotized intercoxal keel in contrast with the fully developed keel of most Elateridae (Fig 3N) and the cantharoid-type ventrite 1, which is simple and without any keel (all Lycidae Lampyridae, Elateridae: *Drilus*; Fig 3R). The terminal abdominal segments resemble those of Elateridae as well as some Lycidae (Fig 2H–2K). Considering the molecular topologies (Fig 1A and 1B) [1–3,8–10,14, 15]), we can conclude that the modifications resulting from incomplete sclerotization are (1) homoplastic and (2) gradual. Therefore, the characters shared by all incompletely sclerotized elateroid families pose a serious problem for phylogenetic inference [32]. We suppose that besides these convergent morphological characters, the loss of apomorphic structures resulted in the inappropriately high rank given to *Plastocerus* in the morphology-based classification.

The sexual dimorphism of *Plastocerus* is limited to the shape of antennae, terminal abdominal ventrites, and slightly wider pronotum (Fig 2A, 2B and 2J). The Crowson's statement [5] on wingless and brachelytrous females of *Plastocerus angulosus* is therefore not correct. The females have fully developed elytra and hind wings. Both male and female genitalia resemble those of Elateridae (Figs 2G, 2N, 2O and 3K). The brachelytrous female was potentially another morphological trait considered by Crowson [5] when Plastoceridae were given a family rank.

The current molecular analyses inferred that *Plastocerus* was in a distant position from other morphologically divergent Elateridae, i.e., Agrypninae: Drilini and Elaterinae: Cebrionini, including *Octinodes* Candèze, 1863, a genus earlier included in Plastoceridae *sensu* Leconte 1861 [16] and Schwarz, 1907[17] (Fig 1B). All these taxa were given the family rank, i.e., families Cebrionidae, Drilidae and Plastoceridae, and were earlier placed in Cantharoidea [5,11,13]. Their high rank was inferred from their morphological divergence when they were compared with fully sclerotized Elateridae. The morphology of *Octinodes* and *Aplastus* Leconte, 1859, both considered to be the close relatives of *Plastocerus* by Leconte [16], was studied by Crowson [5] and no morphological character was found that supported their close relationships. Similarly, the molecular phylogeny (Fig 1) shows that these lineages are distantly related. Hence, the distant position of cebrionid click beetles and *Plastocerus* is simultaneously supported by the current molecular and earlier morphological analyses [5,6] and is not discussed further here.

### Classification of soft-bodied and neotenic beetles

The monophyly of higher taxa and classification based exclusively on phylogenetic relationships are the basic principles of biological classification. Moreover, the monophyletic taxa can be defined only by synapomorphies [33]. If the taxon-defining synapomorphies are lost and incomplete sclerotization affects distantly related lineages in a similar way, we should not use only morphological traits and should apply molecular data as an independent character system.

There is a long list of superfamily- and family-rank taxa grouped by morphological similarities, which in fact are a result of incomplete sclerotization and therefore later refuted when such knowledge was obtained. Historically, soft-bodied beetles were placed in Malacodermata [11], but this taxon was dissolved when the first rigorous morphology-based analyses were conducted [12]. Cantharoidea, either as a superfamily [5] or as a cantharoid clade [4,6,27], gradually shrank when some soft-bodied lineages, e.g., Dascillidae: Karumiinae and Byrrhoidea: Cneoglossidae, were excluded [5,13]. Finally, the cantharoid clade was rejected by molecular studies [1–3,8–10,14,15]. Similarly, numerous soft-bodied taxa with the family-rank were down-ranked and redefined. The taxonomic units (e.g., family) containing unrelated soft-bodied lineages were repeatedly refuted, e.g., the extremely wide concepts of Drilidae and Omalisidae, which included unrelated genera now classified in Lampyridae, Elateridae and Lycidae [5,34]. Several taxa were given high ranks due to the absence of the apomorphic structures seen in close relatives and morphological uniqueness [4,5,13]. For example, the family Podabrocephalidae (type-genus *Podabrocephalus* Pic, 1913) was placed in Elateroidea incertae sedis [4,13] until McKenna *et al*. [2] provided the molecular evidence that *Podabrocephalus* is closely related to fully sclerotized Ptilodactylidae (Elateriformia: Byrrhoidea), and the family was down-ranked to a subfamily in that family [19]. As a further example of a deep conflict between morphology and molecular phylogeny, we refer to Drilini (Agrypninae), formerly a family in Cantharoidea or Elateroidea but recently recovered as a terminal click-beetle lineage [2,3,8,14,15]. Similarly, Telegeusidae Leng, 1920 were down-ranked to Telegeusinae in Omethidae [8]. The morphologically modified *Thylodrias contractus* represented a previously independent subfamily in Dermestidae [35] but was lately down-ranked to the tribe Thylodriini in Trinodinae based on larval morphology [36].

These examples show that *Plastocerus* is not the only distinct elateroid that has been down-ranked and transferred between families. As a similar variability in modifications can be identified in the unrelated lineages of incompletely sclerotized beetles [8,10,37–40], we suggest that these gradual modifications represent a continuous spectrum from soft-bodied-ness to neotenic larviform females. Collectively, we can designate these modifications as an incomplete metamorphosis, and they can be a result of simple modifications in the endocrine system [41,42]. The resulting gradually modified and highly homoplastic morphological traits are therefore inadequate for diagnosing high-rank taxa without an additional independent source of evidence.

## Supporting information

S1 Supplements (PDF)
