# Supplementary material for: Incomplete sclerotization and phylogeny: The phylogenetic classification of Plastocerus (Coleoptera: Elateroidea)
Source: PLoS One. 2018 Mar 14;13(3):e0194026. doi: 10.1371/journal.pone.0194026 (PMC5851614; doi:10.1371/journal.pone.0194026)
Supplement: S1 Supplements — (PDF) [file pone.0194026.s001.pdf]

Incomplete sclerotization and phylogeny: the phylogenetic classification of *Plastocerus* (Coleoptera: Elateroidea)

Ladislav Bocak, Michal Motyka, Matej Bocek, Milada Bocakova

Supplementary Table A. The list of taxa and GenBank Accession Numbers for the Elateroidea and Elateridae datasets.

**Outgroups**			*SSU*	*LSU*	*rrnL*	*cox1*	Voucher
Buprestidae	Agrilinae	*Agrilus* sp.	AF451934	DQ198701	DQ198622	DQ198544	UPOL 001047
Buprestidae	Buprestinae	*Anthaxia hungarica*	DQ100484	DQ198702	DQ198623	DQ198545	UPOL 000M24
Byrrhidae	Byrrhinae	*Byrrhus pilula*	AF427604	DQ198705	DQ198625	DQ198548	BMNH 679172
Byrrhidae	Syncalyptinae	*Chaetophora spinosa*	AF451929	DQ198706	-	-	BMNH 679203
Byrrhidae	Syncalyptinae	*Curimopsis setigera*	AF451930	DQ198707	-	-	BMNH 679204
Callirhipidae	-	gen. sp.	KF625511	KF626112	KF625818	KF625211	UPOL 001249
Callirhipidae	-	gen. sp.	DQ100490	DQ198726	DQ198637	DQ198560	UPOL 000M23
Chelonariidae	-	gen. sp.	KF625509	KF626110	KF625817	KF625210	UPOL 001324
Chelonariidae	-	gen. sp.	DQ100488	DQ198724	DQ198635	DQ198558	UPOL 000M06
Clambidae	-	gen. sp.	KF625497	KF626105	KF625804	KF625196	UPOL 001320
Clambidae	Clambinae	*Clambus pubescens*	EF362951	-	AM884186	DQ155704	BMNH 673260
Dermestidae	Orphilinae	*Orphilus subnitidus*	KP419204	KP419560	-	-	MSC1282
Dermestidae	Orphilinae	*Orphilus niger*	EF213882	EF213915	EF213848	-	BMNH 667510
Dermestidae	Dermestinae	*Dermestes peruvianus*	AY748111	DQ202647	DQ202548	DQ221956	BMNH 669240
Dermestidae	Dermestinae	*Dermestes laniarius*	EF213892	EF213923	EF213860	EF213947	BMNH 677523
Dermestidae	Dermestinae	*Dermestes murinus*	EF213875	-	EF213831	DQ155790	BMNH 677490
Dermestidae	Dermestinae	*Dermestes* sp. A	KP419077	KP419430	-	-	BT0061
Dermestidae	Dermestinae	*Dermestes undulatus*	-	EF213907	EF213838	-	BMNH 677499
Dermestidae	Dermestinae	*Dermestes* sp. B	-	JN097697	JN097782	-	WKU-KLB-12
Dermestidae	Attageninae	*Attagenus pelio*	AY748110	-	DQ202544	-	BMNH 679216
Dermestidae	Attageninae	*Attagenus* sp.	HQ419173	-	-	HQ419107	MC2010-80
Dermestidae	Trinodinae	*Trinodes rufescens*	EF213891	EF213922	EF213859	EF213946	BMNH 677522
Dermestidae	Trinodinae	*Trinodes hirtus*	EF362968	-	-	-	BMNH 724082
Dermestidae	Megatominae	*Anthrenus verbasci*	AY748112	-	-	HM243383	BMNH 679151
Dermestidae	Megatominae	*Anthrenus* cf. *lepidus*	KP419005	-	-	-	DDM0374
Dermestidae	Megatominae	*Anthrenus scrophulariae*	EF213894	EF213924	EF213863	EF213948	BMNH 677525
Dermestidae	Megatominae	*Anthrenus* sp.	-	-	JN097742	JN097704	WKU-KLB-4
Dermestidae	Megatominae	*Anthrenocerus* sp.	HQ419172	-	-	HQ419106	MC2010
Dermestidae	Megatominae	*Trogoderma* sp. A	HQ419152	-	-	HQ419083	MC2010-107
Dermestidae	Megatominae	*Trogoderma* sp. B	HQ419175	-	-	HQ419109	MC2010-97
Dermestidae	Megatominae	*Trogoderma granarium*	HQ419157	-	KJ930431	HQ419090	-
Dermestidae	Megatominae	*Megatoma* sp.	-	-	JN097781	-	WKU-KLB-13
Dryopidae	Dryopinae	*Pomatinus substriatus*	AF451924	DQ198708	DQ198626	DQ198549	BMNH 693616
Eulichadidae	-	*Eulichas* sp.	DQ100489	DQ198725	DQ198636	DQ198559	UPOL 000M22

Heteroceridae	Heterocerinae	*Heterocerus* sp.	AF451928	DQ198718	Q198630	DQ198553	UPOL 001048
Hydraenidae	Ochthebiinae	*Ochthebius minimus*	HE970955	HE970995	HE971074	HE970917	MNCN AI347
Hydraenidae	Ochthebiinae	*Ochthebius melanescens*	AJ810732	AJ810767	-	HE970900	MNCN AI344
Hydrophilidae	Georissinae	*Georissus crenulatus*	AY745584	DQ202637	DQ202580	DQ221983	BMNH 679200
Hydrophilidae	Sphaeridiinae	*Cercyon ustulatus*	AM287129	-	AM287071	AM287093	174909
Hydrophilidae	Chaetarthriinae	*Anacaena globulus*	AM287125	-	AM287064	AM287086	-
Hydrophilidae	Hydrophilinae	*Hydrobius fuscipes*	AJ810720	-	AM287070	AM287092	-
Psephenidae	Eubrianacinae	*Eubrianax* sp.	DQ100485	DQ198721	DQ198632	DQ198555	UPOL 000M33
Psephenidae	-	gen. sp.	KF625514	KF626115	KF625820	KF625214	UPOL RK0168
Psephenidae	-	gen. sp.	KF625515	KF626116	KF625821	KF625215	UPOL RK0169
Ptilodactylidae	Ptilodactylinae	*Ptilodactyla serricornis*	AF451932	DQ198723	DQ198634	DQ198557	BMNH 693606
Ptilodactylidae	Cladotominae	*Paralichas pectinatus*	DQ100486	DQ198722	DQ198633	DQ198556	UPOL 000M41
Ptilodactylidae	-	gen. sp.	KF625517	KF626118	KF625823	KF625222	UPOL RK0131
Ptilodactylidae	-	gen. sp.	KF625518	KF626119	KF625824	KF625217	UPOL RK0149
Ptilodactylidae	-	gen. sp.	KF625520	KF626121	KF625825	KF625219	UPOL RK0151
Ptilodactylidae	-	gen. sp.	KF625521	KF626122	KF625826	KF625220	UPOL RK0152
Scarabaeidae	Aphodiinae	*Australam. occidentalis*	EF487639	AY132457	EF487822	EF656781	BMNH 703639
Scarabaeidae	Sericinae	*Serica brunnea*	EF487712	EU084263	EF487872	EF487776	BMNH 703005
Scarabaeidae	Melolonthinae	*Lepidiota stradbrokensis*	EF487696	EU084209	EF487881	EF487763	BMNH 671319
Scarabaeidae	Sericinae	*Gynaecoserica variipennis*	EF487683	EU084189	EF487968	EF487752	BMNH 678396
Scarabaeidae	Melolonthinae	*Holotrichia seticollis*	EF487687	DQ524596	DQ680877	DQ524528	BMNH 677874
Scarabaeidae	Cetoniinae	*Oxythyrea cinctella*	EF487653	EU084149	EF487962	EF487733	BMNH 678461
Scarabaeidae	Scarabaeinae	*Cheironitis hoplosternus*	AY821528	AY131781	AY131597	AY131940	BMNH 679878
Scarabaeidae	Aphodiinae	*Podotenus storeyi*	EF487648	AY132494	EF487788	AY132432	BMNH 703575
Scarabaeidae	Dynastinae	gen. sp.	EF487663	AY132488	EF487817	AY132397	BMNH 703635
Scarabaeidae	Sericinae	*Omaloplia nigromarginata*	EF487705	EU084255	EF487791	EF487770	BMNH 747063
Scarabaeidae	Scarabaeinae	*Onthophagus crinitis*	AY821535	AY131759	AY131574	AY131924	BMNH 679858
Scirtidae	Scirtinae	*Cyphon hilaris*	AF201419	DQ198698	DQ198620	DQ198542	BMNH 679123
Scirtidae	Scirtinae	*Scirtes hemisphericus*	AF451937	DQ198699	-	-	BMNH 679275
Scirtidae	-	gen. sp.	KF625498	KF626098	KF625807	KF625199	UPOL RK0147
Scirtidae	Scirtinae	*Scirtes* sp.	KF625500	KF626100	KF625808	KF625201	UPOL RK0160
Scirtidae	Scirtinae	*Cyphon* sp.	KF625501	KF626101	KF625809	KF625202	UPOL RK0161
Scirtidae	-	gen. sp.	KF625502	KF626102	KF625810	KF625203	UPOL RK0162
Scirtidae	-	gen. sp.	KF625504	KF626104	KF625812	KF625205	UPOL RK0165
Silphidae	Silphinae	*Oiceoptoma thoracicum*	AJ810736	AB285581	AB285549	AB606436	-
Sphaeritidae	Sphaeritinae	*Sphaerites glabratus*	AJ810728	DQ202650	AM287077	DQ222001	BMNH 679280
Staphylinidae	Pseudopsinae	*Pseudopsis sulcata*	AY745630	DQ202651	DQ202587	DQ221990	BMNH 679246
Staphylinidae	Scaphidiinae	*Scaph. quadrimaculatum*	AY745631	DQ202643	DQ202582	DQ221985	BMNH 679234
Staphylinidae	Oxytelinae	*Bledius femoralis*	AY745627	DQ202681	DQ202608	DQ222015	BMNH 679369
Ingroup							
Artematopodidae	Artematopodinae	*Eurypogon brevipennis*	KF294763	KF294769	KF294757	KF294776	UPOL 001335
Artematopodidae	Artematopodinae	*Eurypogon japonicus*	KF294761	KF294767	KF294755	KF294774	UPOL RK0091

Artematopodidae	Artematopodinae	*Eurypogon hisamatsui*	KF294762	KF294768	KF294756	KF294775	UPOL RK0128
Cantharidae	Malthininae	*Inmalthodes* sp.	KF625603	KF626205	KF625895	KF625303	UPOL 001289
Cantharidae	Silinae	gen. sp.	KF625608	KF626210	KF625900	KF625307	UPOL 001294
Cantharidae	Silinae	gen. sp.	KF625609	KF626211	KF625901	KF625308	UPOL 001295
Cantharidae	Chauliognathinae	*Chauliognathus* sp.	KF625613	KF626215	KF625905	KF625312	UPOL 001299
Cantharidae	Cantharinae	*Athemellus insulsus*	KF625620	KF626222	KF625912	KF625319	UPOL 001306
Cantharidae	Chauliognathinae	gen. sp.	KF625623	KF626225	KF625915	KF625322	UPOL 001309
Cantharidae	Malthininae	gen. sp.	KF625626	KF626228	KF625918	KF625325	UPOL 001312
Cantharidae	Cantharinae	gen. sp.	KF625629	KF626231	KF625921	KF625328	UPOL 001315
Cantharidae	Chauliognathinae	gen. sp.	KF625635	KF626237	KF625927	KF625334	UPOL 001394
Cantharidae	Cantharinae	*Rhagonycha lignosa*	AF451939	DQ198770	DQ198687	DQ198610	BMNH 679176
Cantharidae	Silinae	*Laemoglyptus* sp.	DQ100528	DQ198771	DQ198688	DQ198611	UPOL 000M10
Cantharidae	Chauliognathinae	*Ichthyurus* sp.	DQ100531	DQ198774	DQ198691	DQ198614	UPOL 000M12
Cantharidae	Malthininae	gen. sp.	DQ100533	DQ198777	DQ198694	DQ198617	UPOL 000M21
Cantharidae	Malthininae	*Inmalthodes* sp.	DQ100534	DQ198778	DQ198695	DQ198618	UPOL 000M27
Cantharidae	Malthininae	*Malthinus* sp.	KF625596	KF626198	KF625890	KF625296	UPOL RK0181
Cantharidae	Chauliognathinae	*Chauliognathus opacus*	HM156710	HM156702	FJ613418	FJ613418	BT0055
Cerophytidae	Cerophytinae	*Cerophytum elateroides*	KF625714	KF626302	KF626002	KF625407	UPOL RK0129
Elateridae	Agrypninae	*Drilus flavescens*	DQ100501	DQ198748	DQ198657	DQ198579	UPOL 001046
Elateridae	Agrypninae	*Agrypnus m*urinus	AF451943	DQ198735	DQ198645	DQ198567	UPOL 001049
Elateridae	-	gen. sp.	KF625762	KF626346	KF626057	-	UPOL 001237
Elateridae	Elaterinae	gen. sp.	KF625763	KF626345	-	KF625459	UPOL 001413
Elateridae	Elaterinae	*Mulsanteus* sp	HQ333744	HQ333839	KF626037	HQ333929	UPOL 001414
Elateridae	Elaterinae	*Anoplischius* sp	HQ333745	HQ333840	HQ333664	HQ333930	UPOL 001415
Elateridae	Agrypninae	*Conoderus* sp	HQ333746	HQ333841	HQ333665	HQ333931	UPOL 001416
Elateridae	Agrypninae	*Conoderus* sp	HQ333747	HQ333842	HQ333666	HQ333932	UPOL 001417
Elateridae	Agrypninae	*Platycrepidius* sp.	HQ333748	HQ333843	HQ333667	HQ333933	UPOL 001418
Elateridae	Elaterinae	*Octinodes* sp.	HQ333749	HQ333844	HQ333668	HQ333934	UPOL 001419
Elateridae	Agrypninae	*Pyrophorus* sp.	HQ333751	HQ333846	HQ333670	HQ333936	UPOL 001421
Elateridae	Agrypninae	*Chalcolepidius* sp.	HQ333752	HQ333847	HQ333671	HQ333937	UPOL 001422
Elateridae	Agrypninae	*Pyrophorus* sp.	HQ333753	HQ333848	HQ333672	KF625435	UPOL 001423
Elateridae	Thylacosterninae	*Balgus* sp.	DQ100500	DQ198746	DQ198655	-	BMNH 669202
Elateridae	Dendrometrinae	*Stenagostus rhombeus*	AF451945	DQ198744	DQ198653	DQ198576	BMNH 679144
Elateridae	Elaterinae	*Ampedus balteatus*	AF427605	DQ198736	DQ198646	DQ198568	BMNH 679173
Elateridae	Dendrometrinae	*Athous haemorrhoidalis*	AF451944	DQ198738	DQ198648	DQ198570	BMNH 679174
Elateridae	Cardiophorinae	gen. sp.	AF451942	DQ198739	DQ198649	DQ198571	BMNH 679341
Elateridae	Elaterinae	*Cebrio* sp.	DQ100497	DQ198740	DQ198650	DQ198572	BMNH 679376
Elateridae	Elaterinae	*Agriotes lineatus*	-	DQ198733	DQ198643	DQ198566	BMNH 703086
Elateridae	Dendrometrinae	*Denticollis linearis*	DQ100498	DQ198741	DQ198651	DQ198573	UPOL 000M25
Elateridae	Dendrometrinae	*Athous vittatus*	HQ333755	HQ333850	HQ333674	HQ333939	UPOL RK0002
Elateridae	Elaterinae	*Agriotes acuminatus*	HQ333756	HQ333851	HQ333675	HQ333940	UPOL RK0003
Elateridae	Agrypninae	*Agrypnus* sp.	HQ333757	HQ333852	HQ333676	HQ333941	UPOL RK0004
Elateridae	Elaterinae	*Ampedus* sp.	HQ333758	HQ333853	HQ333677	HQ333942	UPOL RK0005

Elateridae	Dendrometrinae	*Denticollis* sp.	HQ333759	HQ333854	KF626041	HQ333943	UPOL RK0006
Elateridae	Elaterinae	*Ampedus sanguinolentus*	HQ333760	HQ333855	HQ333678	HQ333944	UPOL RK0007
Elateridae	Dendrometrinae	*Anostirus purpureus*	HQ333761	HQ333856	HQ333679	HQ333945	UPOL RK0008
Elateridae	Elaterinae	*Ampedus rufipennis*	HQ333762	HQ333857	HQ333680	HQ333946	UPOL RK0009
Elateridae	Dendrometrinae	*Nothodes parvulus*	HQ333763	HQ333858	HQ333681	KF625443	UPOL RK0010
Elateridae	Cardiophorinae	*Dicronychus rubripes*	HQ333764	HQ333859	KF626023	HQ333947	UPOL RK0011
Elateridae	Elaterinae	*Elater* sp.	HQ333766	HQ333861	HQ333683	HQ333949	UPOL RK0013
Elateridae	Elaterinae	gen. sp.	HQ333767	HQ333862	HQ333684	HQ333950	UPOL RK0014
Elateridae	Agrypninae	*Cryptalaus* sp.	HQ333768	HQ333863	HQ333685	HQ333951	UPOL RK0015
Elateridae	Elaterinae	gen. sp.	HQ333769	HQ333864	HQ333686	HQ333952	UPOL RK0016
Elateridae	Elaterinae	gen. sp.	HQ333770	HQ333865	HQ333687	HQ333953	UPOL RK0017
Elateridae	Elaterinae	*Ampedus* sp.	HQ333771	HQ333866	HQ333688	HQ333954	UPOL RK0018
Elateridae	Elaterinae	gen. sp.	HQ333773	HQ333868	HQ333690	HQ333956	UPOL RK0020
Elateridae	Dendrometrinae	*Selatosomus latus*	HQ333774	HQ333869	HQ333691	HQ333957	UPOL RK0021
Elateridae	Dendrometrinae	*Pheletes quercus*	HQ333775	HQ333870	HQ333692	HQ333958	UPOL RK0022
Elateridae	Cardiophorinae	*Dicronychus cinereus*	HQ333776	HQ333871	KF626024	HQ333959	UPOL RK0023
Elateridae	Elaterinae	*Ludioschema* sp.	HQ333777	HQ333872	HQ333693	HQ333960	UPOL RK0024
Elateridae	Agrypninae	*Adelocera* sp.	HQ333778	HQ333873	HQ333694	HQ333961	UPOL RK0025
Elateridae	Elaterinae	gen. sp.	HQ333780	HQ333875	KF626038	HQ333963	UPOL RK0027
Elateridae	Agrypninae	*Cryptalaus* sp.	HQ333781	HQ333876	KF626034	-	UPOL RK0028
Elateridae	Agrypninae	*Agrypnus* sp.	HQ333783	HQ333878	HQ333697	HQ333965	UPOL RK0030
Elateridae	Cardiophorinae	gen. sp.	HQ333784	HQ333879	KF626025	HQ333966	UPOL RK0031
Elateridae	Elaterinae	*Priopus ornatus*	HQ333785	HQ333880	HQ333698	HQ333967	UPOL RK0032
Elateridae	Elaterinae	*Agriotes ustulatus*	HQ333786	HQ333881	HQ333699	HQ333968	UPOL RK0033
Elateridae	Dendrometrinae	gen. sp.	HQ333787	HQ333882	HQ333700	HQ333969	UPOL RK0034
Elateridae	Cardiophorinae	gen. sp.	HQ333788	HQ333883	KF626026	HQ333970	UPOL RK0035
Elateridae	Cardiophorinae	*Cardiophorus erichsoni*	HQ333790	HQ333885	HQ333701	HQ333972	UPOL RK0037
Elateridae	Elaterinae	*Ampedus sinuatus*	HQ333791	HQ333886	HQ333702	HQ333973	UPOL RK0038
Elateridae	Dendrometrinae	*Cidnopus pilosus*	HQ333792	HQ333887	HQ333703	HQ333974	UPOL RK0039
Elateridae	Agrypninae	*Drasterius bimaculatus*	HQ333793	HQ333888	HQ333704	HQ333975	UPOL RK0040
Elateridae	Agrypninae	*Adelocera* sp.	HQ333794	HQ333889	KF626035	HQ333976	UPOL RK0041
Elateridae	Dendrometrinae	*Hypolithus* sp.	HQ333795	HQ333890	HQ333705	HQ333977	UPOL RK0042
Elateridae	Negastriinae	*Zorochros* sp.	HQ333796	HQ333891	HQ333706	HQ333978	UPOL RK0043
Elateridae	Dendrometrinae	*Semiotus* sp.	HQ333799	HQ333894	HQ333709	HQ333981	UPOL RK0046
Elateridae	Dendrometrinae	*Oxynopterus* sp.	HQ333800	HQ333895	HQ333710	HQ333982	UPOL RK0047
Elateridae	Elaterinae	gen. sp.	HQ333801	HQ333896	HQ333711	HQ333983	UPOL RK0048
Elateridae	Negastriinae	*Quasimus* sp.	HQ333802	HQ333897	HQ333712	HQ333984	UPOL RK0049
Elateridae	Negastriinae	*Quasimus* sp.	HQ333803	HQ333898	HQ333713	HQ333985	UPOL RK0050
Elateridae	Elaterinae	*Anchastus* sp.	HQ333804	HQ333899	HQ333714	HQ333986	UPOL RK0051
Elateridae	Elaterinae	*Agriotes obscurus*	HQ333805	HQ333900	KF626039	KF625441	UPOL RK0052
Elateridae	Agrypninae	*Adelocera* sp.	HQ333806	HQ333901	HQ333715	HQ333987	UPOL RK0053
Elateridae	Elaterinae	*Ludioschema* sp.	HQ333808	HQ333903	HQ333717	HQ333989	UPOL RK0055
Elateridae	Elaterinae	*Anchastus* sp.	HQ333809	HQ333904	HQ333718	HQ333990	UPOL RK0056

Elateridae	Agrypninae	*Agrypnus* sp.	HQ333810	HQ333905	HQ333719	HQ333991	UPOL RK0057
Elateridae	Elaterinae	gen. sp.	HQ333812	HQ333907	HQ333720	HQ333993	UPOL RK0059
Elateridae	Elaterinae	*Anchastus* sp.	HQ333813	HQ333908	HQ333721	HQ333994	UPOL RK0060
Elateridae	Elaterinae	*Anchastus* sp.	HQ333814	HQ333909	HQ333722	HQ333995	UPOL RK0061
Elateridae	Agrypninae	*Adelocera* sp.	HQ333815	HQ333910	KF626033	KF625439	UPOL RK0062
Elateridae	Agrypninae	*Drasterius* sp.	HQ333816	HQ333911	HQ333723	HQ333996	UPOL RK0063
Elateridae	Agrypninae	*Adelocera* sp.	HQ333817	HQ333912	HQ333724	HQ333997	UPOL RK0064
Elateridae	Agrypninae	*Adelocera* sp.	HQ333818	HQ333913	HQ333725	HQ333998	UPOL RK0065
Elateridae	Elaterinae	gen. sp.	HQ333819	HQ333914	HQ333726	HQ333999	UPOL RK0066
Elateridae	Agrypninae	*Agrypnus* sp.	HQ333820	HQ333915	HQ333727	HQ334000	UPOL RK0067
Elateridae	Elaterinae	*Priopus humeralis*	HQ333821	HQ333916	HQ333728	HQ334001	UPOL RK0068
Elateridae	Elaterinae	*Ampedus* sp.	HQ333822	HQ333917	HQ333729	HQ334002	UPOL RK0069
Elateridae	Cardiophorinae	gen. sp.	HQ333823	HQ333918	HQ333730	HQ334003	UPOL RK0070
Elateridae	Agrypninae	*Selasia* sp.	HQ333824	HQ333919	HQ333731	HQ334004	UPOL RK0071
Elateridae	Agrypninae	*Drilus* sp.	HQ333826	HQ333921	HQ333733	HQ334006	UPOL RK0073
Elateridae	Agrypninae	*Drilus* concolor	HQ333827	KF626322	HQ333734	HQ334007	UPOL RK0074
Elateridae	Lissominae	*Drapetes mordelloides*	HQ333828	HQ333922	HQ333735	HQ334008	UPOL RK0075
Elateridae	Agrypninae	*Drilus* sp.	-	-	HQ333739	HQ334013	UPOL RK0081
Elateridae	Agrypninae	*Cryptalaus* sp.	HQ333834	HQ333926	HQ333740	HQ334014	UPOL RK0082
Elateridae	Agrypninae	*Drilus mauritanicus*	HQ333837	HQ333928	HQ333743	HQ334016	UPOL RK0085
Elateridae	Elaterinae	*Cebrio* sp.	KF625745	KF626329	KF626040	KF625440	UPOL RK0142
Elateridae	Agrypninae	*Selasia* sp.	KF625739	KF626323	KF626027	KF625433	UPOL RK0158
Elateridae	Agrypninae	*Selasia* sp.	KF625740	KF626324	KF626028	KF625434	UPOL RK0172
Elateridae	Lissominae	gen. sp.	KF625760	KF626336	KF626051	KF625450	UPOL RK0174
Elateridae	Elaterinae	gen. sp.	KF625752	KF626337	KF626052	KF625449	UPOL RK0203
Elateridae	Agrypninae	*Pyrophorus* sp.	KF625742	KF626325	KF626030	KF625436	UPOL RK0213
Elateridae	Agrypninae	*Chalcolepidius* sp.	KF625743	KF626326	KF626031	KF625437	UPOL RK0219
Elateridae	Dendrometrinae	gen. sp.	KF625751	KF626338	KF626053	KF625451	UPOL RK0251
Elateridae	Agrypninae	gen. sp.	KF625761	KF626341	KF626055	KF625452	UPOL RK0301
Elateridae	Elaterinae	*Octinodes* sp.	KF625746	KF626328	-	KF625442	UPOL RK0306
Elateridae	Elaterinae	gen. sp.	KF625758	KF626334	KF626056	KF625453	UPOL RK0310
Elateridae	Lissominae	gen. sp.	KF625747	KF626330	KF626042	KF625444	UPOL RK0334
Elateridae	Lissominae	gen. sp.	KF625748	KF626331	KF626043	KF625445	UPOL RK0335
Elateridae	Lissominae	gen. sp.	KF625749	KF626332	KF626044	KF625446	UPOL RK0353
Elateridae	Lissominae	gen. sp.	KF625750	KF626333	KF626045	KF625447	UPOL RK0354
Elateridae	Agrypninae	*Malacogaster passerini*	KF625741	KF626321	KF626029	KF625432	UPOL RK0369
Elateridae	Elaterinae	gen. sp.	KF625755	KF626339	KF626046	KF625455	UPOL RK0400
Elateridae	Hemiopinae	gen. sp.	KF625756	KF626342	KF626047	KF625456	UPOL RK0403
Elateridae	Elaterinae	gen. sp.	KF625757	KF626343	KF626048	KF625457	UPOL RK0404
Elateridae	Elaterinae	gen. sp.	KF625759	KF626344	KF626049	KF625458	UPOL RK0406
Elateridae	Plastocerinae	*Plastocerus angulosus*	KX648442	KX648444	KX648440	KX648446	UPOL A01544
Elateridae	Dendrometrinae	*Aplotarsus incanus*	-	DQ198737	DQ198647	DQ198569	BMNH 703088
Elmidae	Elminae	*Limnius volckmari*	AF451914	DQ198712	DQ198627	DQ198550	BMNH 679263

Elmidae	Larainae	*Potamophilus acuminatus*	AF451911	-	EF209464	EF209584	EC_E07
Eucnemidae	Melasinae	*Micorhagus pygmaeus*	KF625570	KF626170	KF625867	KF625271	UPOL 001224
Eucnemidae	-	gen. sp.	KF625571	KF626171	KF625868	KF625272	UPOL 001225
Eucnemidae	-	gen. sp.	KF625572	KF626172	KF625869	KF625273	UPOL 001226
Eucnemidae	Macraulacinae	gen. sp.	KF625578	KF626178	KF625873	KF625276	UPOL 001233
Eucnemidae	Macraulacinae	gen. sp.	KF625580	KF626180	KF625875	KF625278	UPOL 001235
Eucnemidae	-	gen. sp.	KF625581	KF626181	KF625876	KF625279	UPOL 001236
Eucnemidae	-	gen. sp.	KF625582	KF626182	KF625877	KF625280	UPOL 001328
Eucnemidae	Macraulacinae	gen. sp.	KF625583	KF626184	KF625879	KF625282	UPOL 001330
Eucnemidae	Macraulacinae	*Nematodes* sp.	DQ100495	DQ198731	DQ198642	DQ198564	BMNH 703107
Eucnemidae	-	gen. sp.	KF625549	KF626149	KF625849	KF625250	UPOL RK0123
Eucnemidae	-	gen. sp.	KF625551	KF626153	KF625853	KF625254	UPOL RK0171
Eucnemidae	-	gen. sp.	KF625557	KF626157	KF625857	KF625258	UPOL RK0343
Eucnemidae	-	gen. sp.	KF625561	KF626161	KF625861	KF625262	UPOL RK0347
Iberobaeniidae	Iberobaeninae	*Iberobaenia minuta*	KT339296	KT339297	-	KT339298	UPOL RK0790
Lampyridae	Luciolinae	gen. sp.	DQ100514	DQ198762	DQ198672	DQ198595	UPOL 000M03
Lampyridae	Luciolinae	*Curtos* sp.	DQ100513	DQ198761	DQ198671	DQ198594	UPOL 000M16
Lampyridae	Ototretinae	*Flabellotreta* sp.	DQ100520	DQ198763	DQ198678	DQ198601	UPOL 000M34
Lampyridae	Ototretinae	gen. sp.	DQ100521	DQ198764	DQ198679	DQ198602	UPOL 000M37
Lampyridae	-	gen. sp.	KF625638	KF626239	KF625929	KF625337	UPOL RK0093
Lampyridae	-	gen. sp.	KF625650	KF626246	KF625941	KF625349	UPOL RK0108
Lampyridae	-	gen. sp.	KF625652	KF626247	KF625943	KF625351	UPOL RK0110
Lampyridae	-	gen. sp.	KF625653	KF626248	KF625944	KF625352	UPOL RK0111
Lampyridae	-	gen. sp.	KF625657	KF626250	KF625948	KF625356	UPOL RK0173
Lampyridae	-	gen. sp.	KF625663	KF626253	KF625954	KF625362	UPOL RK0378
Lampyridae	-	gen. sp.	KF625668	KF626257	KF625959	KF625367	UPOL RK0383
Lampyridae	-	gen. sp.	KF625670	KF626259	KF625961	KF625369	UPOL RK0385
Lampyridae	-	gen. sp.	KF625675	KF626262	KF625966	KF625374	UPOL RK0390
Lampyridae	-	gen. sp.	KF625677	KF626264	KF625968	KF625376	UPOL RK0392
Lampyridae	-	gen. sp.	KF625678	KF626265	KF625969	KF625377	UPOL RK0393
Leiodidae	Cholevinae	*Catops picipes*	AJ810734	-	FM209287	FM209288	-
Lycidae	Lycinae	*Plateros* sp.	DQ181059	DQ181133	DQ180985	DQ181207	UPOL 000031
Lycidae	Ateliinae	*Dilophotes* sp.	DQ181066	DQ181140	DQ180992	DQ181214	UPOL 000244
Lycidae	Dictyopterinae	*Dictyoptera elegans*	DQ181073	DQ181147	DQ180999	DQ181221	UPOL 000570
Lycidae	Dictyopterinae	*Dictyoptera speciosa*	DQ181074	DQ181148	DQ181000	DQ181222	UPOL 000571
Lycidae	Dictyopterinae	*Benibotarus spinicoxis*	DQ181076	DQ181150	DQ181002	DQ181224	UPOL 000573
Lycidae	Lycinae	*Platycis nasutus*	DQ181079	DQ181153	DQ181005	DQ181227	UPOL 000576
Lycidae	Lycinae	gen. sp.	DQ181092	DQ181166	DQ181018	DQ181240	UPOL 000592
Lycidae	Lycinae	*Thonalmus hubbardi*	DQ181094	DQ181168	DQ181020	DQ181242	UPOL 000595
Lycidae	Metriorrhynchinae	*Metriorrhynchus lineatus*	-	-	-	DQ904323	UPOL 000829
Lycidae	Libnetinae	*Libnetis* sp.	DQ181104	DQ181178	DQ181030	DQ181252	UPOL 001002
Lycidae	Libnetinae	*Libnetis* sp.	DQ181105	DQ181179	DQ181031	DQ181253	UPOL 001008
Lycidae	Lycinae	*Metapteron* sp.	AF451946	DQ198757	-	DQ198588	BMNH 679218

Lycidae	Lycinae	*Lycus* sp.	DQ181039	DQ181113	DQ180965	DQ181187	UPOL 000L03
Lycidae	Lyropaeinae	*Lyropaeus* sp.	DQ181042	DQ181116	DQ180968	DQ181190	UPOL 000L11
Lycidae	Lycinae	*Dihammatus* sp.	DQ181043	DQ181117	DQ180969	DQ181191	UPOL 000L12
Lycidae	Lycinae	*Calochromus* sp.	DQ181047	DQ181121	DQ180973	DQ181195	UPOL 000L16
Lycidae	Lycinae	*Lyponia nigrohumeralis*	DQ181048	DQ181122	DQ180974	DQ181196	UPOL 000L17
Lycidae	Lycinae	*Macrolycus* sp.	DQ181049	DQ181123	DQ180975	DQ181197	UPOL 000L18
Lycidae	Lycinae	*Microtrichalus* sp.	DQ181052	DQ181126	DQ180978	DQ181200	UPOL 000L23
Lycidae	Lycinae	*Calopteron* sp.	DQ181053	DQ181127	DQ180979	DQ181201	UPOL 000L25
Lycidae	Lycinae	*Lycostomus* sp.	DQ181055	DQ181129	DQ180981	DQ181203	UPOL 000L27
Lycidae	Lycinae	*Conderis* sp.	DQ350139	DQ350138	DQ350141	DQ350140	UPOL 000M42
Lycidae	Ateliinae	*Paratelius nigricornis*	HM451139	HM451095	HM451014	HM451053	UPOL VM0020
Omalisidae	Omalisinae	*Omalisus fontisbellaquei*	AF451948	DQ198749	DQ198658	DQ198580	UPOL 000377
Omalisidae	Thilmaninae	*Pseudeuanoma* sp.	HQ333832	KF626300	HQ333738	HQ334011	UPOL RK0079
Omethidae	Telegeusinae	*Telegeusis nubifer*	DQ100503	DQ198751	DQ198660	DQ198582	UPOL 000321
Omethidae	Driloniinae	*Drilonius* sp.	KF625526	KF626127	KF625835	KF625226	UPOL RK0362
Omethidae	Driloniinae	*Drilonius striatulus*	KF625527	KF626128	KF625830	KF625227	UPOL 001272
Omethidae	Driloniinae	*Drilonius* sp.	KF294764	KF294770	KF294758	KF294777	UPOL 001273
Omethidae	Driloniinae	*Drilonius* sp.	KF625528	KF626129	KF625831	KF625228	UPOL 001274
Omethidae	Omethinae	*Troglomethes leechi*	KF625529	KF626130	KF625828	KF625229	UPOL 001340
Omethidae	Matheteinae	*Ginglymocladus* sp.	KF625530	KF626131	KF625829	KF625230	UPOL 001341
Omethidae	Driloniinae	*Drilonius* sp.	DQ100502	DQ198750	DQ198659	DQ198581	UPOL 000M26
Omethidae	Driloniinae	*Drilonius* sp.	KF625523	KF626124	KF625832	KF625223	UPOL RK0132
Omethidae	Driloniinae	*Drilonius* sp.	KF294765	KF294771	KF294759	KF294778	UPOL RK0134
Omethidae	Driloniinae	*Drilonius* sp.	KF625524	KF626125	KF625833	KF625224	UPOL RK0135
Omethidae	Driloniinae	*Drilonius* sp.	KF625525	KF626126	KF625834	KF625225	UPOL RK0136
Omethidae	Telegeusinae	gen. sp.	KF625531	KF626132	-	KF625231	UPOL RK0360
Phengodidae	Phengodinae	*Phengodes* sp.	KF625725	KF626312	KF626011	KF625418	UPOL 001238
Phengodidae	Phengodinae	*Phengodes* sp.	KF625726	KF626313	KF626012	KF625419	UPOL 001241
Phengodidae	-	gen. sp.	KF625727	KF626314	KF626013	KF625420	UPOL 001243
Phengodidae	-	gen. sp.	KF625728	KF626315	KF626014	KF625421	UPOL 001245
Phengodidae	-	gen. sp.	KF625729	KF626316	KF626015	KF625422	UPOL 001246
Phengodidae	-	gen. sp.	KF625730	KF626317	KF626016	KF625423	UPOL 001247
Phengodidae	-	gen. sp.	KF625736	KF626320	KF626020	KF625430	UPOL 001351
Phengodidae	Phengodinae	*Phengodes* sp.	DQ100504	DQ198752	DQ198661	DQ198583	UPOL 000M29
Phengodidae	-	gen. sp.	KF625719	KF626306	KF626007	KF625412	UPOL RK0361
Phengodidae	-	gen. sp.	KF625721	KF626308	KF626008	KF625414	UPOL RK0364
Phengodidae	Phengodinae	*Phengodes* sp.	KF625723	KF626310	KF626009	KF625416	UPOL RK0366
Phengodidae	-	gen. sp.	KF625724	KF626311	KF626010	KF625417	UPOL RK0368
Rhagophthalmidae	-	gen. sp.	KF625717	KF626305	KF626005	KF625410	UPOL 001359
Rhagophthalmidae	-	*Mimoochotyra* sp.	DQ100505	DQ198753	DQ198662	DQ198584	UPOL 000M30
Rhagophthalmidae	-	gen. sp.	DQ100506	DQ198754	DQ198663	DQ198585	UPOL 000M31
Rhagophthalmidae	Rhagophthalminae	*Bicladodrilus* sp.	DQ100507	DQ198755	DQ198664	DQ198586	UPOL 000M35
Rhagophthalmidae	-	gen. sp.	KF625715	KF626303	KF626003	KF625408	UPOL RK0088

Rhagophthalmidae	-	gen. sp.	KF625716	KF626304	KF626004	KF625409	UPOL RK0370
Rhagophthalmidae	Rhagophthalminae	*Rhagophthalmus ohbai*	AB298864	-	NC010964	NC010964	71225
Throscidae	-	gen. sp.	KF625543	KF626143	KF625842	KF625243	UPOL 1326
Throscidae	Throscinae	*Trixagus dermestoides*	AF451950	DQ198747	DQ198656	DQ198578	BMNH 679235
Throscidae	-	gen. sp.	KF625533	KF626134	KF625836	KF625233	UPOL RK0138
Throscidae	Throscinae	*Trixagus meybohmi*	KF294766	KF294772	KF294760	KF294779	UPOL RK0139
Throscidae	-	gen. sp.	KF625535	KF626136	KF625837	KF625235	UPOL RK0141
Throscidae	-	gen. sp.	KF625536	KF626137	KF625838	KF625236	UPOL RK0175
Throscidae	-	gen. sp.	KF625539	KF626139	KF625839	KF625239	UPOL RK0333
Throscidae	-	gen. sp.	KF625541	KF626141	KF625840	KF625241	UPOL RK0337

References

Bocak *et al*. (2016) The discovery of Iberobaeniidae (Coleoptera: Elateroidea): a new family of beetles from Spain, with immatures detected by environmental DNA sequencing. *Proceedings of the Royal Society of London B–Biological Sciences*, **283**, 20152350.

Bocakova *et al.* (2007) Molecular phylogenetics of Elateriformia (Coleoptera): evolution of bioluminescence and neoteny. *Cladistics*, **23**, 477–496.

Bell, K.L. & Philips, T.K. (2012) Molecular systematics and evolution of the Ptinidae (Coleoptera: Bostrichoidea) and related families. *Zoological Journal of the Linnean Society*, **165**, 88–108

Castalanelli, M. A. et al. (2012) Molecular phylogeny supports the paraphyletic nature of the genus Trogoderma (Coleoptera: Dermestidae) collected in the Australasian ecozone. Bulletin of Entomological Research, **102**, 17–28.

Kundrata, R. & Bocak, L. (2011) The phylogeny and limits of Elateridae (Insecta, Coleoptera): is there a common tendency of click beetles to soft-bodiedness and neoteny? *Zoologica Scripta*, **40**, 364–378.

Kundrata *et al*. 2014 (2014) The comprehensive phylogeny of the superfamily Elateroidea (Coleoptera: Elateriformia). *Molecular Phylogenetics and Evolution*, **76**, 162–171.

Hunt, T. *et al*. (2007) A comprehensive phylogeny of beetles reveals the evolutionary origins of a superradiation. *Science* **318**, 1913–1916.

Hunt, T. & Vogler, A.P. (2008). A protocol for large-scale rRNA sequence analysis: towards a detailed phylogeny of Coleoptera. *Molecular Phylogenetics and Evolution*, **47**, 289–301.

McKenna, D.D. *et al*. (2015) The beetle tree of life reveals that Coleoptera survived end-Permian mass extinction to diversify during the Cretaceous terrestrial revolution. *Systematic Entomology*, **40**, 835–880.

Olson, R.L.O. *et al*. Molecular identification of *Trogoderma granarium* (Coleoptera:Dermestidae) using the 16S gene (unpublished).

Supplementary Table B. Partitioning and models selection of Elateroidea dataset.

Name	Type	Seqs	Sites	Patterns	Constant Sites	Model
rrnL	DNA	183	971	659	51%	TIM2+F+R6
SSU	DNA	186	2492	1116	64%	GTR+F+R6
LSU	DNA	184	1219	736	54%	SYM+I+G4
cox1	CODON	185	241	241	0%	GY+F+G4

Input data: 186 taxa with 4 partitions and 4923 total sites (0.6% missing data)

Supplementary Table C. Partitioning and models selection of Elateridae dataset.

Name	Type	Seqs	Sites	Patterns	Constant Sites	Model
rrnL	DNA	107	707	420	52%	TPM3u+F+I+G4
SSU	DNA	107	1906	261	90%	TIMe+I+G4
LSU	DNA	109	706	232	77%	TIM2e+I+G4
cox1	CODON	107	241	241	0%	GY+F+G4

Input data: 110 taxa with 4 partitions and 3560 total sites (2.4% missing data)

Supplementary Table D. Results of likelihood test constrained trees vs unconstrained tree.

Topology	logL	p-KH	c-ELW	p-AU	Significantly worse tree?
Unconstrained	-110933.919	0.9915	0.9892	0.9918	No
Lycidae+*Plastocerus*	-111010.127	0.0012	0.0007	0.0014	Yes
Cantharidae+*Plastocerus*	-110985.904	0.0085	0.0053	0.0155	Yes
Omethidae+*Plastocerus*	-111049.389	0.0001	0.0000	0.0030	Yes
Lampyridae+*Plastocerus*	-110986.265	0.0083	0.0048	0.0164	Yes

The list of tests (abbreviations):
p-KH: p-value of one sided Kishino-Hasegawa test (1989); c-ELW: Expected Likelihood Weight (Strimmer & Rambaut 2002); p-AU: p-value of approximately unbiased (AU) test (Shimodaira, 2002).
